# The Speciesism Debate: Intuition, Method, and Empirical Advances

**DOI:** 10.3390/ani9121054

**Published:** 2019-12-01

**Authors:** Jeroen Hopster

**Affiliations:** Institut für Philosophie, University of Graz, Heinrichstrasse 26/V, 8010 Graz, Austria; jeroen.hopster@uni-graz.at

**Keywords:** speciesism, intuition, evolutionary debunking arguments, moral psychology, species-relativism, cumulative culture, Peter Singer, Shelly Kagan, Bernard Williams

## Abstract

**Simple Summary:**

An influential idea in animal ethics is that moral favouritism towards members of one’s own species is a prejudice. This prejudice has been labelled ‘speciesism’, in analogy with racism and sexism. But not all ethicists subscribe to the view that speciesism is a prejudice. In fact, the tenability of speciesism is a topic of ongoing ethical debate. A recent exchange between Peter Singer and Shelly Kagan might leave the impression that this debate has essentially reached a stalemate, since the disputing parties rely on irreconcilable moral intuitions. In the present article, I argue that this impression is misleading. I highlight both philosophical and empirical research avenues that can help to move the speciesism debate forward, emphasizing that not all ethical intuitions about speciesism should be given equal weight. The article is part of the special issue ‘Animal Ethics: Questioning the Orthodoxy’.

**Abstract:**

This article identifies empirical, conceptual and normative avenues to advance the speciesism debate. First, I highlight the application of Evolutionary Debunking Arguments (EDAs) as one such avenue: especially where (anti-)speciesist positions heavily rely on appeals to moral intuition, and EDAs have potential to move the debate forward. Second, an avenue for conceptual progress is the delineation of speciesism from other views in its vicinity, specifically from the view that biological differences between species are sometimes morally relevant (‘species-relativism’). Third, if we adopt Singer’s definition of speciesism, then a limitation of the current debate is that it is not obvious whether the core ethical principle that underlies anti-speciesist positions—the *Principle of Equal Consideration of Interests*—is widely applicable. Arguably, the interests of animals are often too dissimilar to establish what *equal* consideration amounts to. I underscore the need for integrating philosophical and empirical research, to come to terms with the extent to which the interests of members of different species are alike, and with the question of whether any dissimilarities might be morally relevant.

*“Our inclination to treat humans as though we are special is no mere prejudice. Despite what Singer says, there is a significant philosophical view at work here—one worthy of careful further investigation.”*

Shelly Kagan [1] (p. 20)

*“At the end of the day, no clear consensus has emerged—or, at best, this one: no rejoinder to Singer’s attack on speciesism has convinced many besides its author, and the challenge remains.”*

François Jaquet [2] (p. 2)

## 1. Introduction

Half a century after the term ‘speciesism’ was coined, the question of whether human beings have an elevated moral status is still a topic of live debate in animal ethics (Appendix A, 1). Commonly taken as a starting point of this debate is Peter Singer’s *Animal Liberation*, where Singer characterizes speciesism as a widespread “prejudice or attitude of bias in favour of the interests of members of one’s own species and against those of members of other species” [3] (p. 6). Singer regards speciesism as discrimination on the basis of species membership, on a par with sexism and racism: speciesists unjustifiably favour the interests of members of their own group over the interests of others.

Singer’s [3,4,5] position regarding speciesism has remained essentially unchanged over the decades, but meanwhile, some prominent ethicists have stood up to challenge his view. For instance, Bernard Williams [6] has argued that ‘being a human’ is itself a morally relevant property, and that a prejudice in favour of human beings can be defended accordingly. More recently, Shelly Kagan [1] has entertained the possibility (without wholeheartedly endorsing it) that a version of ‘personism’ is morally defensible, arguing that *either* personism should be regarded as a speciesist view, *or* most people are not speciesists, *pace* Singer (Appendix A, 2). But new arguments in support of Singer’s position have been articulated too, such as François Jaquet’s [2] Evolutionary Debunking Argument against speciesism, on the basis of findings from experimental moral psychology.

The speciesism debate has several dimensions, and the aforementioned authors are by no means the only voices in the debate. But, in what follows, my discussion will give specific attention to their contributions, as they provide a good impression of the state-of-the-art as well as some of its limitations. My aim will be to articulate certain correctives to the debate and to provide suggestions that can help to advance it. For instance, Kagan’s [1] discussion might leave the impression that the speciesism debate has essentially reached a stalemate since the disputing parties rely on irreconcilable moral intuitions. This impression is misleading, I will argue, and merits to be corrected. One avenue that can help to reinvigorate the debate is research on the origins and justification of moral intuitions—and preliminary findings give reason to believe that on the topic of speciesism, not all ethical intuitions should be given equal weight.

The article proceeds as follows. In Section 2, I introduce Singer’s view of speciesism and highlight the core ethical principle on which it relies: the *Principle of Equal Consideration of Interests.* I defend the principle from Kagan’s recent criticism, specifically from his suggestion that speciesists can simply counter it by appealing to countervailing intuitions. As I underscore in Section 3, not all moral intuitions are created equal: appeals to moral intuition are not warranted if they can be undercut by so-called Evolutionary Debunking Arguments (EDAs). Although further research is called for, preliminary findings from moral psychology suggest that *at least some* speciesist intuitions are proper targets of EDAs. In Section 4, I go on to argue that while empirical research—specifically, research on the question of whether ‘the folk’ endorses speciesism—can help to advance the ethical debate, such research faces various challenges and pitfalls that researchers have to overcome. In Section 5, I return to Singer’s *Principle of Equal Consideration of Interests*, pointing out that it may potentially be of limited scope: if the interests of animals are different in kind, then it is not straightforward what giving these interests *equal* consideration amounts to. In Section 6, I argue that a failure to appreciate this point, might lead to the conflation of two debates: the debate of whether there exists a *moral hierarchy* between members of different species (i.e., the speciesism debate) and the debate of whether there are *morally relevant differences* between the interests of different animals. Building especially on the latter debate, in Section 7, I raise the question of whether there are morally relevant differences between the interests of humans and non-human animals. More specifically, I entertain the proposal—which should be subject to further empirical and normative scrutiny—that the culturally scaffolded lives humans lead have a transformative effect on human interests, in the sense that these interests typically (though not always) differ in kind from the interests of non-human animals. In conclusion, I address a common thread running through my discussion: while both further philosophical analysis and further empirical studies can help to advance the speciesism debate, key advances rely on the interplay between them.

## 2. Singer’s Anti-Speciesist Position: A Defence 

There are various definitions of speciesism in circulation in the academic literature (contrast [1,2,7]) and beyond. Some authors treat speciesism as an unjustified position by definition [8]. This is problematic, however, since the defensibility of speciesism is subject to substantive debate [9]. A more fruitful approach is to distinguish the descriptive concept of speciesism from its normative evaluation. Here, and in what follows, I will adopt Singer’s [3] definition, according to which speciesism involves *the preferential consideration of the interests of members of one’s own species* (Appendix A, 3). 

In this section I outline Singer’s normative argument against speciesism and defend it from a recent criticism by Kagan [1]. Singer’s argument departs from the ethical premise that if the interests of beings are alike, then they should be given equal consideration. For example, if two beings suffer from famine, then their respective interests in avoiding such suffering should weigh equally in our moral decision-making. Importantly, *equal consideration* need not amount to *equal treatment*. Members of different species have different needs and preferences, and as a result differential treatment is often warranted. Obviously, alleviating famine calls for different kinds of treatment, depending on the dietary needs of the starving animal in question. However, and this is Singer’s key point, while differential treatment of animals may be warranted, *preferential consideration* on the basis of species-membership is not. If interests are alike, then it amounts to bias to give preferential consideration to the interests of members one’s own species. Any additional properties that members of the human species might have are morally irrelevant, if the interests of human and non-human animals are alike, Singer argues:

“Can they suffer? Can they enjoy life? If so, they have interests that we should take into account, and we should give those interests equal weight with the interests of all other beings with similar interests. We should not discount their interests in not *suffering* because they cannot talk or because they are incapable of reasoning; and we should not discount their interests in enjoying life, in having things that are fulfilling and rewarding for them, either. The principle of equal consideration of interests should apply to both humans and animals. That’s the sense in which I want to elevate animals to the moral status of humans.”

[4] (p. 575)

To recapitulate, the core ethical principle underlying Singer’s anti-speciesist position can be spelled out as follows.

• ***Principle of Equal Consideration of Interests:* We must give equal weight to like interests**

We have good grounds for endorsing the *Principle of Equal Consideration of Interests* as a weighty ethical principle since it embellishes moral values that many people hold in high regard: fairness and non-discrimination. Indeed, it is the same principle that underlies ethical condemnation of racism and sexism. Singer regards these attitudes as homologous: if we submit that racism and sexism are morally wrong, then by the same token, we should submit that speciesism is wrong.

Although Singer does not employ the term, we can understand his analogy between speciesism on the one hand and racism and sexism on the other, as an application of *consistency reasoning* [10]. The basic idea of consistency reasoning is that like cases should be treated alike. If cases are not treated alike, then there should be a *morally relevant difference* between them. Ethical reasoning typically proceeds in this manner, and justifiably so: consistency reasoning seems to be an appropriate method for reasoning with moral cases [11]. 

Accordingly, disagreement in ethics typically amounts to disagreement over which properties should be regarded as morally relevant. Such disagreement also exists in the speciesism debate. Contra Singer, some authors maintain that there is a morally relevant difference between preferential consideration on the basis of race or sex and on the basis of species membership (Appendix A, 4). For instance, Kagan [1] (p. 8) argues that while in the contexts of racism or sexism preferential consideration is based on false empirical claims, in the context of speciesism it is based on a justifiable appeal to moral intuition. One way to spell out this intuition, Kagan proposes, is to argue that the ‘ownership’ of a given experience is a morally relevant property [1] (p. 5). For example, when considering the interest in ‘not experiencing pain’, one might regard it as morally relevant *who* experiences pain. If *ownership of pain* makes a morally relevant difference, then the pain of human and non-human animals need not be regarded as being of equal moral weight. Accordingly, speciesists may find the following principle intuitively plausible. 

• ***Principle of Morally Relevant Ownership:* It is morally relevant whether or not an interest belongs to a human**

Let us suppose, for the sake of argument, that this principle adequately captures speciesists’ intuitions. The principle entails that the interests of a human and non-human animal in ‘not experiencing pain’ should *not* be given equal consideration. Hence, the principle is incompatible with Singer’s *Principle of Equal Consideration of Interests.* Which of these two principles to adopt? Kagan submits that Singer offers no argument to debunk a speciesist principle along these lines; he merely relies on an appeal to his own countervailing principle. This appeal, however, is ultimately grounded in intuition, Kagan argues, just like the speciesist’s appeal to the *Principle of Morally Relevant Ownership* is. There seem to be no further grounds for favouring Singer’s anti-speciesist intuition over the speciesist’s intuition. Hence, Kagan’s diagnosis leaves the impression that we are faced with a clash of moral intuitions, leading to a stand-off in the speciesism debate.

In the remainder of this section, as well as the next one, I will argue that this impression is misleading: even if speciesist and anti-speciesist positions ultimately rest on irreconcilable intuitions about which properties count as morally relevant, it would be a mistake to think that these intuitions should necessarily be given equal weight. One reason why this is a mistake, I will argue in Section 3, has to do with the different epistemic statuses of intuitions: if we know that a given moral intuition comes about through a process that typically gives rise to cognitive biases, then this detracts from the plausibility of the intuition. Moreover, while further research is called for, there is preliminary experimental evidence that *at least some* speciesist intuitions indeed come about through processes typically associated with cognitive biases [2,7] (Appendix A, 5).

But with regard to the apparent stand-off between Singer and the speciesist view imagined by Kagan, there are also straightforward ethical considerations that can help to adjudicate the debate. Consider which of the principles each party advances is better supported by well-established moral values and principles. As noted, Singer’s principle is plausible on ethical grounds since it embellishes the values of fairness and non-discrimination. By contrast, it is far from obvious that Kagan’s principle gets much support from our moral background knowledge. The principle embellishes the values of partiality and favouritism, which we typically disvalue from an ethical point of view. Moreover, Kagan does not clarify why a racist or sexist could not advance a similar principle, arguing, say, that it is morally relevant whether or not a given experience is ‘owned’ by a white man. The unwelcome association with sexism and racism (cf. [5]), combined with the fact that Kagan’s principle seems to lack positive support from our moral background knowledge, gives us good reason to discard it and endorse Singer’s principle instead. 

## 3. Speciesist Intuitions and Evolutionary Debunking Arguments

On Kagan’s analysis, conflicting moral intuitions play a key role in the speciesism debate. Consider once more Kagan’s argument for taking seriously the speciesist view that ownership of pain is morally relevant. While Kagan himself does not wholeheartedly endorse this view, he does regard it as a moral intuition that should be taken seriously:

“Admittedly, I have offered no argument for [this] speciesist view. Perhaps the claim that human suffering counts more is simply an intuition that some people have, nothing more. But even if so, that hardly shows there is anything wrong with the view.”

[1] (p. 6)

Indeed, Kagan maintains that speciesists are entitled to rely on the strongly held intuition that ownership of pain is morally relevant, even without giving this intuition much argumentative support, because Singer similarly relies on a moral intuition when suggesting that morally relevant interests are grounded in *sentience*:

“[O]ne would be hard pressed to think of anything other than intuition to support the claim that the line between sentience and nonsentience is a morally significant one. So Singer himself is going to have to admit that the appeal to intuition carries force in questions like these. And once he has done that, it seems he should admit that it is just as legitimate for the speciesist to appeal to her intuition that the line between humans and animals is also a morally significant one, in which case speciesism is no more a mere prejudice than sentientism!”

[1] (p. 7)

But do these intuitions have similar argumentative standing, as Kagan suggests? Findings from cognitive psychology indicate that our intuitions on some topics are hardly reliable [12]. In the field of ethics, too, the reliability of intuitions has recently become a topic of debate, specifically in the context of Evolutionary Debunking Arguments. Roughly speaking, EDAs are arguments that aim to show that the causal explanation (e.g., the evolutionary aetiology) of a normative belief detracts from the belief’s justification. Applied to moral intuitions, EDAs typically purport to show that the causal origins of intuitions make them unreliable. Drawing on Kahane’s [13] (p. 106) discussion, they have the following structure:

(1) Our moral intuition that P is explained by X.
(2) X is an off-track process (i.e., a process that is not conducive towards tracking P’s truth).
(3) Therefore, our moral intuition that P is unjustified.

EDAs hold a promise to revitalise existing ethical debates, especially debates that appear to have turned into deadlocks between conflicting moral intuitions [2]. Instead of discrediting some moral intuitions by appealing to other, more plausible moral intuitions, as in the discussion at the end of Section 2, EDAs can be invoked to scrutinize the tenability of moral intuitions on external grounds—i.e., in terms of their epistemic reliability. By way of example, consider Kelly’s [14] dismissal of moral intuitions, which are triggered by feelings of disgust. The mechanisms at the heart of the psychological disgust system originally evolved to protect us from harmful food and parasites. Feelings of disgust were later co-opted in our moral psychology. But arguably, these feelings are not well-attuned to the moral domain: they are sensitive to influences that tend to lead our moral judgements off-track. If this is correct, then moral intuitions that are triggered by a disgust response should be regarded as generally unreliable.

Now consider once again Kagan’s appeal to speciesist intuitions. Giving speciesist intuitions presumptive weight is justified, Kagan argues, because these intuitions are widely held; moreover, Singer’s anti-speciesist intuitions are given presumptive weight just as well. But this argument can only be sustained if the widely held intuitions Kagan appeals to are not ‘off-track’, and equally reliable as Singer’s intuitions. It is not obvious that this is the case: in fact, there are reasons to think that untutored speciesist intuitions are a particularly promising target for EDAs. This is because speciesist intuitions, just like racist and sexist intuitions (which are common targets of EDAs in applied ethics), are self-serving: having such intuitions typically benefits the individual having them, irrespective of their truth or justification. Indeed, preliminary evidence from psychology suggests that similar mechanisms underlie both speciesism and other forms of prejudice: there is a positive correlation between speciesism and prejudicial attitudes such as racism, sexism, and homophobia [7].

Examining the potential of EDAs is a promising avenue for further research on the topic of speciesism. Other preliminary findings on this topic come from Jaquet, who presents evidence that what he dubs “the speciesist belief” results from the cognitive dissonance of meat-eaters, and who argues that these origins detract from the belief’s reliability [2]. This is an interesting claim, and Jaquet’s combined normative and experimental approach provides a good example of how inquiry on these matters can proceed. But more work remains to be done. As I will discuss in Section 6, unfortunately, Jaquet operationalizes “the speciesist belief” with a definition that diverges from Singer’s. As a result, his argument does not speak directly to the debate between speciesists and anti-speciesists in Singer’s sense. Moreover, the success of Jaquet’s EDA hinges on the question of the extent to which eating meat is the *principal* reason for why people endorse speciesism. Jaquet’s findings do not settle this question; while the “meat paradox” may certainly be *one* of the reasons, it is difficult to estimate its weight. Doing so will require further investigation into the nature of people’s moral views about speciesism. 

## 4. Researching ‘Folk Speciesism’: Six Caveats

The question of the extent to which ‘the folk’ endorses speciesism, and of people’s rationales for endorsing speciesism, is currently under-researched [7]. Further research on these questions is an important direction to advance the speciesism debate. Conducting this research, however, as well as interpreting it, is less straightforward than might appear at first. In this section, I highlight six caveats and interpretative difficulties that come along with experiments on folk speciesism and provide suggestions to foster methodological and conceptual progress.

First, one should be careful not to overstate the normative significance of research on folk belief. What if it turns out that a great majority of people are indeed speciesists? Surely, this does not suffice to corroborate speciesism as the correct ethical view. Instead, the most important normative purpose for which experimental findings about folk opinion can be put to use is more indirect: it can help to evaluate the moral reliability of speciesist intuitions. For instance, suppose that research demonstrates that speciesist intuitions are quick and cursory, whereas anti-speciesist intuitions only emerge after sustained reflection (cf. [15]). Arguably, this would raise doubts about the moral tenability of speciesist intuitions. Or consider Caviola et al.’s [7] argument, which suggests that speciesism is psychologically related to specific forms of prejudice. Arguably, this finding strengthens the ethical case for anti-speciesism.

Second, however, a major caveat that complicates assessments of the ethical relevance of extant work on this topic is that different authors tend to define speciesism in different ways. This is illustrated by the few existing studies that explicitly focus on testing to what extent people endorse speciesist intuitions, which operationalize speciesism somewhat differently from each other [2,7], and altogether differently from Singer’s [3] definition.

Third, while folk opinion is often scrutinized by means of survey studies, in such studies the subtleties of different ethical positions are easily lost. Consider the following possibility: while *prima facie* it may appear that respondent A is a speciesist, respondent A actually subscribes to the *Principle of Equal Consideration of Interests* but thinks that this principle only rarely applies, since the interests of different animals are typically not sufficiently similar (see Section 5 for further discussion). Technically, in this case, we should not regard respondent A as a speciesist, at least not in Singer’s sense. However, there is reason to think that in experimental studies, this technicality will easily be overlooked, since it is also sometimes overlooked in discussions by philosophical experts (see Section 6).

Fourth, in survey research, respondents’ immediate responses are typically probed by means of a forced choice paradigm (see [16] for discussion). When this method is used, researchers are likely to capture people’s *first-off* intuitions, rather than their *tutored* intuitions. While for some purposes, (e.g., in advancing EDAs) people’s *first-off* intuitions might be more telling than their tutored intuitions, this is not always the case. For instance, when philosophers make a claim from the armchair about the contents of folk intuition, they might intend to make a claim about such intuitions under idealized conditions—for example, as they would come out in Socratic dialogue. It should be kept in mind that such claims cannot be straightforwardly corroborated or debunked on the basis of survey-based research. 

Fifth, we should bear in mind that apart from professional ethicists, few people have ever explicitly reflected on the relevant ethical principles that play a role in the speciesism debate. This further complicates the question of whether people are normatively committed to speciesism (which is distinct from the question of whether people *behave as if they are*). Given a lack of previous familiarity with the relevant ethical principles, research questionnaires might easily give rise to a training component, whereby respondents *update* their ethical views.

Sixth, we should not assume from the outset that folk opinion will cluster into any clear majority position. It is quite possible that opinions on the issue of speciesism radically diverge. Researchers should take the latter possibility seriously and not preclude it by presenting their respondents with restrictive questionnaires. Ideally, to acquire a balanced view of folk opinion, survey-based studies should be complemented with methods of dialogue and reflection [17]. 

## 5. Limitations of Singer’s Anti-Speciesist Position

In the previous sections, I have argued that Singer’s anti-speciesist position is ethically plausible and that although further research is needed, there is preliminary reason to think that his position can get further support from EDAs. But in the present section, I will argue that Singer’s *Principle of Equal Consideration of Interests* also faces a challenge, which may serve to limit its scope. Recall that according to this principle, we must give equal weight to like interests. As Kagan [1] (p. 5) points out, much hinges on the latter qualification—interests should be *alike*. We might call this the principle’s *Similarity Condition*: 

• ***Similarity Condition:* The interests of the individuals to whom the Principle of Equal Consideration of Interests applies should be sufficiently alike**

The *Similarity Condition* should be fulfilled for Singer’s principle to be applicable. It might be argued, however, that the interests of members of different species are rarely sufficiently alike to allow for equal consideration. Instead, animal interests across species are typically *qualitatively different*. If this is correct, then giving unequal consideration to these interests need not amount to a speciesist prejudice, *sensu* Singer. After all, his principle is compatible with the view that if interests are sufficiently dissimilar, they need not be given equal consideration.

To illustrate this, consider how experiences of suffering might differ across species (Appendix A, 6). Homo sapiens is a markedly social species, with high levels of parental bonding. As a result, human individuals typically suffer immensely from the loss of a child. Also, humans have highly developed cognitive capacities, and can, therefore, suffer from cognitively sophisticated emotions, such as the emotion of regret. Now consider the green sea turtle, which is a solitary species whose members do not invest in parental care, and are unlikely to suffer as a result of cognitively sophisticated emotions like regret. As a result, the kinds of suffering that a human and a green sea turtle experience may be quite different, and their interests in not-suffering are extremely difficult to compare. Obviously, these differences justify differential treatment, which is unproblematic on Singer’s account. But more worrisome, the differences also seem to preclude equal consideration of interests, since the respective interests are simply too dissimilar to establish what *equal* consideration would amount to (of course, the dissimilarity does not preclude consideration of interests *as such*). Under such conditions, Singer’s *Principle of Equal Consideration of Interests* cannot straightforwardly be applied.

Before we can legitimately apply Singer’s principle, then, we first have to examine to what extent suffering in different species is similar. Or put in terms of interests: to what extent are their ‘interests in not suffering’ sufficiently alike? This is a complicated question. It becomes even more complicated when we switch focus from interests regarding *not suffering*, to interests regarding *enjoying life*, or general evaluations of *quality of life* [18]. Plausibly, evaluations of quality of life will be rather diverse across species, and difficult to compare between them. But this difficulty notwithstanding, people seem to be prone to make such comparisons anyway. Experimental results suggest that species-relative considerations about quality of life play an important role in ordinary moral deliberation [19] (p. 58). Moreover, such considerations also figure explicitly in the reasoning of professional ethicists—notably in Singer’s own reasoning, when he argues that:

“pain and *suffering* are equally bad—and pleasure and happiness equally good—whether the being experiencing them is human or non-human, rational or non-rational, capable of discourse or not. On the other hand, death is a greater or lesser loss depending on factors like the extent to which the being was aware of his or her existence over time, and of course the quality of life the being was likely to have, had it continued to live.”

[4] (p. 576)

A plausible interpretation of Singer’s view is as follows: he maintains that interests closely associated with a capacity for sentience (such as suffering, pain and pleasure) can be compared fairly straightforwardly, but he also grants that in other respects, the interests of different animals may differ and that these differences preclude application of the *Principle of Equal Consideration of Interests*. Indeed, as the quoted passage suggests, Singer even submits that what might *prima facie* appear to be similar interests—such as an interest in not dying—can ultimately turn out to be distinct interests, depending on the self-awareness and temporal extension of the animals whose interests they are.

The upshot of our examination of the *Principle of Equal Consideration of Interests*, then, is as follows. While this principle is plausible on ethical grounds (Section 2), there is preliminary reason to think that it has limited application. It may well turn out that only in a limited number of cases, the interests of different animals are sufficiently similar to apply the principle. If the *Similarity Condition* is not met, then as far as the principle goes, we might legitimately give animal interests differential concern (Appendix A, 7). As a result, there might be ample contexts in which we can give special consideration to the interests of members of our own species—and we can do so without being speciesists, in Singer’s sense.

This analysis also suggests a future research avenue that will be particularly important for the speciesism debate. Such research involves both a strong philosophical and a strong empirical component. A crucial philosophical task is to normatively evaluate when the interests of different animals count as sufficiently similar. A crucial empirical task is to investigate, in detail, the experiences of members of different species, such as the experience of suffering. The latter task brings along a philosophical challenge as well: apart from behavioural observations, it requires researchers to integrate knowledge from different fields and make plausible conjectures about animal phenomenology (cf. [20]). Close collaboration between philosophers and empirical scientists is called for.

## 6. Speciesism versus Species-Relativism: Disentangling Two Debates

As pointed out in the previous section, Singer’s anti-speciesist position is compatible with the view that there are morally relevant differences between the interests of members of different species (Appendix A, 8). This point is sometimes lost on contributors to the speciesism debate. For instance, as noted in Section 3, François Jaquet ([2] *passim*) defines “the speciesist belief” as “the belief that there is a morally relevant difference between humans and other animals.” (Appendix A, 9) Thus defined, however, almost all contemporary ethicists—including Singer—should be classified as speciesists. Since such a classification would be bizarre, it seems natural to conclude that Jaquet’s definition misses the mark (Appendix A, 10).

This confusion points to a distinction between two orthogonal debates about the moral relevance of species membership, which is sometimes overlooked in the literature: a debate between speciesists and anti-speciesists, and a debate between species-relativists and species-egalitarians. The former debate is essentially about the question of whether there exists a *moral hierarchy* between animals of different species. Sociological studies [21], as well as experimental findings [19], suggest that people tend to give preferential consideration to members of some species over others. Is people’s invocation of a ‘sociozoological scale’ morally justifiable? Are some animals indeed *more significant* from a moral point of view than others? Do they rank *higher* on the moral ladder, and deserve *better* moral treatment? **Speciesists** answer affirmatively, at least where our own species is concerned: we should favour the interests of humans, even when the interests of other species are alike. **Anti-speciesists**, on the other hand, subscribe to the *Principle of Equal Consideration of Interests*: if the interests of animals are alike, then equal consideration is called for.

The other debate is essentially about whether there are *morally relevant differences* between animals of different species. **Species-relativists** answer affirmatively: there are morally relevant differences between members of different species, in virtue of which their interests may justifiably be given differential consideration. But differential consideration need not amount to *favouritist* consideration. It is perfectly possible that a species-relativist is also an anti-speciesist, and maintains that if interests are alike, they should be given equal consideration. However, as it happens, interests are often not alike—and especially where humans are concerned, the differences are morally relevant. (Appendix A, 11) This position stands in opposition to a position that has been labelled species-egalitarianism, according to which all species have equal moral standing and command equal respect ([22]; see [23] for critical discussion). **Species-egalitarians** maintain that while animals (as well as plants) of different species are dissimilar, these dissimilarities are morally irrelevant: all living beings deserve equal moral consideration (Appendix A, 12).

Species-egalitarianism and anti-speciesism are orthogonal positions; the former entails the latter, but the latter does not entail the former. Adherents of both positions endorse the aforementioned *Principle of Equal Consideration of Interests*: 

• ***Principle of Equal Consideration of Interests:* We must give equal weight to like interests**

But species-egalitarians subscribe to an additional principle, which is *incompatible* with species-relativism: 

• ***Principle of No Morally Relevant Differences:* There are no morally relevant differences between species**

A useful way to distinguish the four abovementioned positions is by making their commitments regarding these two moral principles explicit:

As Table 1 brings out, a commitment to speciesism is more distinctive than a commitment to species-relativism, and a commitment to species-egalitarianism is more distinctive than a commitment to anti-speciesism. The middle positions are more generic and partly overlap: Singer, for instance, is both a species-relativist and an anti-speciesist.

In closing this section, let’s look at a possible way of specifying the position of the species-relativist in some more detail. As outlined above, species-relativists maintain that there are morally relevant differences between members of different species. However, this still leaves open whether species-membership is what *grounds* these differences, or whether the differences are grounded in some other property. For instance, what might be morally relevant according to species-relativists is not species-membership itself, but certain *capacities* of animals, which happen to *correlate* strongly with species membership (cf. [24] (p. 184)). 

While this ‘correlational’ version of species-relativism might seem plausible at first glance, it may be problematic on closer scrutiny. Suppose we grant the species-relativist that there are morally relevant differences between members of different species. There is reason to believe that if there are such differences, and if they are not grounded in species-membership, then they also *do not* strongly correlate with species membership. After all, the capacities that are the most obvious candidates for justifying differential moral treatment—for example, whether animals belong to a prey or predator species, are social or solitary, have high or low investment in parental care, produce a lot or little offspring—are shared by a vast number of species. Hence, biological species-membership seems much too narrow as a category to strongly correlate with morally relevant properties. Consider, for example, that on biological classifications, the ‘Sumatran orangutan’ and the recently discovered ‘Tapanuli orangutan’ should be regarded as different species [25], whereas there are barely any perceivable differences between them—certainly no differences that anyone would regard as morally relevant. The differentiation between these species is made predominantly on the basis of genetics. By contrast, properties that species-relativists are likely to regard as morally relevant are behavioural traits and mental capacities, or perhaps relational traits such as phylogenetic proximity to our own species [21] (Appendix A, 13).

It may, of course, be the case that because of some historical happenstance (e.g., because our closest sister-species have gone extinct), Homo sapiens is somewhat atypical in this regard, and that humans possess a set of morally relevant features that *does* uniquely correlate with species membership. However, this is not obviously true, as suggested by the fact that several of the capacities that have been hailed as being characteristic of ‘persons’, such as self-awareness, psychological unity, and temporal continuity, might be shared by several species (e.g., [26]) (Appendix A, 14). Nevertheless, in the next and final section, I will argue that the correlational version of species-relativism still holds some promise. Homo sapiens may indeed be atypical: arguably, as a contingent matter of fact, there are certain morally relevant features about the lives of human beings, which are largely absent from the lives of non-human animals.

## 7. The Case for Human Exceptionalism: Dead, Loose, and Open Ends

Thus far, in discussing speciesism, we have specifically looked at views that privilege the interests of certain animals on the basis of their *individual* characteristics. This is commonplace in animal ethics: many authors—including Singer—subscribe to the view of ‘moral individualism’, according to which the particular characteristics of individuals, rather than group membership, should be the determinant of our moral consideration. [24] (p. 173)

But even if commonplace, it is not obvious that this view is correct. Detractors might argue that group membership can also be regarded as a morally relevant property. Consider the fact that conservationists commonly ascribe a specific normative status to a given population of animals (e.g., ‘to be protected’). This status, in turn, has normative implications for the way that individuals who are members of this population ought to be treated. Naturally, it could be questioned whether the practice of granting different conservation statuses to different groups of animals is appropriate: the fact that we engage in this practice does not imply that the practice is justified. But the fact that this practice is commonplace and fairly uncontroversial provides preliminary reason to think that appeals to the moral significance of group membership are at least sometimes justified.

In this section, I first discuss a way of advancing a ‘non-individualist’ speciesist view in more detail by considering an argument put forward by Bernard Williams. I argue that his speciesist view is either a dead end or insufficiently developed: Williams ultimately *insists on* the moral relevance of group-membership (more specifically, on the relevance of “being a human”), rather than *justifying* it. But I go on to propose that a slightly different argument, which blurs the boundaries of ‘moral individualism’ and ‘non-individualism’, holds more promise: arguably, the corollaries of collective human behaviour are significant for appreciating the morally relevant features of individual human lives. 

First, consider Williams’ [6] view. According to Williams, ethics is an unavoidably human project, and our ethical concepts are shaped accordingly. (Appendix A, 15) Williams illustrates this by presenting the thought-experiment of an invasion by intelligent but disgusting aliens, who threaten to take over our planet and exterminate our species. In response, he imagines that humans defend their cultural and ethnic identity with appeals to “defend humanity” and “stand up for human beings.” He remarks:

“This is an ethical appeal in an ethical dispute. (…) The relevant ethical concept is something like: loyalty to, or identity with, one’s ethnic or cultural grouping. Moreover—and this is the main lesson of this fantasy—this is an ethical concept we already have. This is the ethical concept that is at work when, to the puzzlement of the critics, we afford special consideration to human beings because they are human beings. (…) So the idea of there being an ethical concept that appeals to our species membership is entirely coherent.”

[6] (p. 150)

In this thought-experiment, Williams singles out a concept entrenched in ordinary thought—the concept of being loyal to one’s own species. But why should we take this concept to have prescriptive ethical force, rather than regarding it as a vestige of outdated wisdom, that ought to be rejected? Surely the fact that we *have* the concept of loyalty to one’s own group does not suffice to establish that the concept’s contents are *morally desirable*. If group-loyalty is just a prejudice, then Williams’ position falls straight within the target of Singer’s criticism: given the *Principle of Equal Consideration of Interests*, it should be discarded.

The weak spot of Williams’ position is that he grants that favouritism towards fellow humans is ultimately indeed *just a prejudice*: we happen to be on the side of humans, rather than any other group (see Singer’s [5] criticism). But perhaps, by granting this, Williams concedes too much and overlooks the possibility that preferential treatment of members of our own group might be justified by virtue of certain features that are unique to our species. Consider the fact that humans are engaged in intergenerational projects of knowledge accumulation, which have led to a rich understanding of ourselves, our surroundings, and our place in the universe—an understanding that is unquestionably much richer than that of any other species we are familiar with (Appendix A, 16). We have knowledge of our own origins and history, we have accumulated a cultural legacy that is unprecedented among life on our planet, and we have erected—and continue to refine—elaborate institutions in domains such as education, healthcare, art, and science, which enable ever more individuals to benefit from our collective, intergenerational achievements. Arguably, these and similar achievements make our species worth preserving (cf. [27])—perhaps more so than other species. If so, then under conditions of pending extinction, as Williams envisions in his thought-experiment, we might be entitled to claim a distinct normative status (‘to be protected’) for members of our own species, without this being *mere prejudice* (Appendix A, 17).

Moreover, arguably, our cumulative cultural achievements have also enriched the lives of *all human individuals* who benefit from them: they make human lives more interesting, diverse, surprising, rich and worthwhile than the lives of other animals, or at least potentially so (Appendix A, 18). Therefore, at least in most (though not all) cases, there is some *prima facie* plausibility to the claim that the quality of the lives of individual humans, understood as a function of their ‘richness’, is quite different from the quality of life of most non-human animals (see also [18] (p. 186)). Of course, this claim needs further substantiation: an adequate defence requires a detailed normative account of what makes a life rich, as well as intimate acquaintance with the lives of the animals who make up the comparison class. Once again, a combination of philosophical and empirical work is called for.

In closing, let’s return to the speciesism debate. Is the view sketched above, according to which the cumulative cultural capacities of our species affect that moral status of human individuals, a speciesist view? That depends. One might argue that our cultural heritage has transformed human lives in morally relevant ways, and that, as a result, the interests of individual humans are typically different from those of other animals, and in many cases too dissimilar to apply the *Principle of Equal Consideration of Interests*. This view would count as ‘species-relativist’, though not necessarily as speciesist in Singer’s sense. A genuinely speciesist view contains an additional normative claim: our unique cultural capacities elevate the moral status of human individuals, in the sense that they make human interests *more significant* than the like interests of other animals. But note that this additional claim does not follow from the suggestion that our cultural achievements have had a transformative effect on human lives; further argument is needed, from those who aspire to defend it.

## 8. Conclusions

There are various ways in which the speciesism debate can be moved forward. A recurring theme in this article has been the importance of integrating empirical research and philosophical analysis. The plausibility of philosophical claims often depends on empirical findings; empirical research, in turn, often relies on tacit conceptual and normative assumptions, which merit philosophical scrutiny. One specific issue where a close interplay between philosophical and empirical work can advance the debate is in scrutinizing the tenability of speciesist intuitions. Ethicists often treat strongly held intuitions as argumentative fixed-points, but the presumptive weight of moral intuitions should not be taken for granted. To what extent people indeed have speciesist intuitions, and whether such intuitions can be successfully targeted by Evolutionary Debunking Arguments, are important questions for future research.

Integrated philosophical and empirical work will also be important in evaluating the applicability of Singer’s *Principle of Equal Consideration of Interests*. I have argued that if animal interests are typically dissimilar, Singer’s principle has limited application. To what extent animal interests are in fact dissimilar, however, is an open question, which requires further empirical research. Additionally, it is a question with a normative component, for it is an ethical question under what conditions the interests of different animals count as sufficiently similar to merit equal consideration. Especially with regard to the interests of human individuals, and the question to what extent these are comparable to the interests of non-human animals, the speciesism debate raises issues that are is still far from resolved.

## Figures and Tables

**Table 1:** Four distinct positions concerning the moral relevance of species-membership.

	Principle of Equal Consideration of Interests	Principle of No Morally Relevant Differences
**Speciesists**	deny	deny
**Anti-Speciesists**	endorse	deny or endorse
**Species-Relativists**	deny or endorse	deny
**Species-Egalitarianists**	endorse	endorse

## Appendix A. Endnotes

1. The term speciesism was coined in 1970 by Richard D. Ryder, in a privately published pamphlet. Peter Singer took the term from Ryder and popularized it in *Animal Liberation* (1975).
2. In philosophical parlance, a ‘person’ is roughly a being that is rational, conscious, self-aware and temporally extended. The version of personism that Kagan [1] proposes as worthy of “careful further investigation” is “modal personism”—the view according to which the metaphysical fact that one *could have been* a person is morally significant.
3. Singer (1975, p. 6), too, evaluates speciesism as “a prejudice or attitude of bias”, thus intertwining a description of speciesism with its evaluation. But as Kagan [1] (p. 2) points out, Singer’s view can be restated in descriptive terms.
4. There are different arguments to this effect (i.e., arguments in defence of speciesism). For instance, some ethicists argue that our relationship to fellow human beings is similar to the special relations we have towards our family members, and which—on some moral views—can justify preferential moral treatment towards them (e.g., Scanlon [28] (p. 185); see McMahan [29] for critical discussion). It is beyond the scope of the current article to discuss all defences of speciesism that have been given in the literature. Here and in what follows, I primarily focus on the defence of speciesism advanced by Kagan (Section 2 and Section 3) and by Williams (Section 7).
5. Additionally, there are also *historical* reasons to think that the association between speciesism and racist prejudice is not without support: the appeals to the biology of present-day speciesists share common origins with the biological appeals of 19th and early 20th century racists [30] (p. 42).
6. The argument that suffering in different species can be qualitatively different is fairly commonplace in the animal ethics literature. For instance, McMahan [31] argues that “the greater psychological depth, complexity, and unity in most human beings make it possible for them to have lives that contain more, and arguably more important, dimensions of the good (such as significant accomplishment, personal relations based on deep mutual understanding, and so on) and are therefore more worth living than those of animals. In most cases, therefore, the psychological damage caused by suffering is worse in human beings because the life that is damaged matters more.”
7. Of course, it should be kept in mind that this need not be the *only* ethically relevant principle in the context of speciesism.
8. Kumar [32] (p. 71) highlights roughly the same point: “It does not follow (…) from species membership not justifying what some take it to justify that species membership is morally irrelevant.”
9. In the article’s abstract “the speciesist belief” is characterized in line with Singer’s formulation, namely as the belief “that human interests matter much more than the like interests of non-human animals” [2] (p. 1). But in the remainder of the article, Jaquet builds on the alternative definition, cited above.
10. Another example of a discussion that conflates these debates can be found in Cohen [33] (p. 867).
11. An example is Nussbaum’s [34] capabilities approach, which proposes a species-specific norm of flourishing as a yardstick for evaluating the flourishing of individual animals.
12. For instance, Taylor [35] (p. 35) argues that “[r]ejecting the notion of human superiority entails its positive counterpart: the doctrine of species impartiality. One who accepts that doctrine regards all living things as possessing inherent worth—the same inherent worth since no one species has been shown to be either higher or lower than any other.”
13. A further problem for species-relativists is the ‘species problem’ in the philosophy of biology, which appears to be unsolvable [36]. Species are difficult to define; on recent counts, between 27 and 92 different ‘species concepts’ have been proposed, many of which are incompatible. If species cannot be clearly defined, then it seems problematic to assert that morally relevant properties strongly correlate with species membership.
14. ‘Species overlap’ regarding capacities that seem to be morally relevant, is one of two considerations that drives a key argument against speciesism, the so-called ‘argument from marginal cases’ (the other consideration is the fact that some humans lack these capacities).
15. A somewhat related view—and defence of speciesism—has been advanced by Cora Diamond [37]. Diamond argues that the concept of being human is not a biological concept, but rather an “ethical” or “imaginative” concept, which deeply influences our ethical thought. An objection that her view faces, however, is that she fails to establish why the significant role that the property of “being human” plays in ordinary ethical thought, and the fact that its application is restricted to members of our own species, is *appropriate*. As McMahan [29] convincingly argues, when push comes to shove, it is unclear exactly what, on Diamond’s view, can be considered as constitutive of “being human”, other than being a member of a given biological species.
16. For elaboration of the importance (and unprecedented scale) of cumulative culture in the evolution of Homo sapiens, see [38].
17. Note that this distinctive normative status need not be *grounded* in species-membership. One might argue that what matters is the continuation of the *projects* our species is engaged in, not the continuation of the *species* itself. If a different species were to evolve with a similar—or improved—capacity to continue these projects, then there would be no reason to give moral preference to our own species. If Williams’ thought-experiment is understood along these lines, then a special plea to “defend humanity” does turn out to be *just a prejudice*.
18. Here the dividing line between ‘moral individualism’ and its ‘non-individualist’ counterpart has been blurred: we focus on the distinctive character of the lives of human individuals, but this distinctive character is a function of the evolution of human societies.
